# Clinical impacts of total parenteral nutrition in hematopoietic stem cell transplantation patients with high nutritional risk

**DOI:** 10.3389/fnut.2024.1495640

**Published:** 2024-12-13

**Authors:** Le Yang, Di Wu, Junting Dai, Huiyi Lv, Miao Li

**Affiliations:** Department of Pharmacy, The Second Affiliated Hospital of Dalian Medical University, Dalian, Liaoning, China

**Keywords:** total parenteral nutrition, hematopoietic stem cell transplantation, high nutritional risk, catheter-related bloodstream infection, liver function

## Abstract

**Background:**

Hematopoietic stem cell transplantation (HSCT) patients often receive consecutive intensive chemotherapy, which can lead to gastrointestinal complications and acute graft-versus-host disease (GVHD), placing patients at high nutritional risk.

**Aim:**

This retrospective study aimed to evaluate the benefits of nutritional support in maintaining nutritional status, reducing weight loss without increasing the incidence of catheter-related bloodstream infections (CRBSI) or liver dysfunction, and improving clinical outcomes in HSCT patients at high nutritional risk.

**Methods:**

A total of 526 patients who underwent HSCT were included in the study. Based on the Nutrition Risk Screening-2002 (NRS-2002) and propensity score matching, 70 patients were assigned to the control group (without parenteral nutrition) and 70 to the enhanced nutrition group (with parenteral nutrition) between 2012 and 2022. We compared data between the two groups at different time points (days 3, 7, 10, and 14 after transplantation and the day before discharge) on the following: (1) effectiveness: weight loss, albumin, and prealbumin levels; (2) safety: incidence of CRBSI and conjugated bilirubin levels; and (3) clinical outcomes: hospital stay duration, rate of rehospitalization, hospitalization costs, and survival rates.

**Results:**

Our results showed that total parenteral nutrition (TPN) effectively mitigated weight loss on days 10 and 14 and the day before discharge, while also improving albumin (33.41 ± 4.57 in the control group, 34.87 ± 4.08 in the TPN group, *p* < 0.05; 33.72 ± 3.52 in the control group, 35.27 ± 4.04 in the TPN group, *p* < 0.05; 34.09 ± 4.44 in the control group, 35.55 ± 3.87 in the TPN group, *p* < 0.05) and prealbumin (245.18 ± 79.94 in the control group, 274.26 ± 86.73 in the TPN group, *p* < 0.05; 233.27 ± 79.57 in the control group, 279.34 ± 80.20 in the TPN group, *p* < 0.01; 247.24 ± 83.29 in the control group, 280.65 ± 100.22 in the TPN group, *p* < 0.05) levels during the same periods. In addition, there were no significant differences in CRBSI incidence or liver function between the non-TPN and TPN groups. Furthermore, the TPN group experienced a shorter length of hospital stay (48.06 ± 13.90 in the control group, 42.13 ± 14.22^*^ in the TPN group, *p* < 0.05) and lower rates of unexpected rehospitalization (37.1% in the control group, 21.4% in the TPN group, *p* < 0.05).

**Conclusion:**

This study demonstrated that effective TPN formulations improved nutritional status, ensured patient safety, and contributed to better clinical outcomes in HSCT patients at high nutritional risk. These findings support the use of nutritional interventions in hematologic malignancy patients receiving induction therapy prior to transplantation.

## 1 Introduction

Hematopoietic stem cell transplantation (HSCT) is a widely used and effective therapeutic approach for hematologic disorders such as acute lymphoblastic leukemia, acute myeloid leukemia, and lymphoma (1, 2). The China Marrow Donor Program (CMDP) reported a significant increase in the number of HSCT cases, rising from 649 in 2012 and up to 14,551 in 2022 (Supplementary material 2). However, HSCT patients undergo high-dose chemotherapy or total body irradiation, which often leads to severe side effects, including intense nausea, vomiting, diarrhea, mucositis, and Graft-versus-host disease (GVHD) (3, 4). These complications can severely hinder oral intake and disrupt the absorption of essential nutrients, profoundly affecting the patient’s nutritional status.

Given these challenges, addressing and managing nutritional concerns in HSCT patients is crucial for mitigating adverse effects and optimizing transplant outcomes (5, 6). Therefore, maintaining adequate nutrition is an urgent priority for patients undergoing HSCT (7). Current guidelines recommend total parenteral nutrition (TPN) for HSCT recipients who are unable to maintain at least 50% of their recommended caloric intake (8). However, the potential drawbacks of TPN, such as an increased risk of catheter-related bloodstream infections (CRBSI) and liver function abnormalities, should not be overlooked as these complications can negatively impact patient outcomes (9).

In children and adolescents treated with high-dose chemotherapy followed by autologous hematopoietic stem cell transplants, parenteral nutrition has been associated with changes in body weight and serum liver function tests compared to those receiving no parenteral nutrition during remission induction treatments (10). These findings highlight the need for a comprehensive assessment of the risks and benefits of TPN. Ensuring a balanced approach that weighs the advantages of nutritional support against the risks of potential complications is essential when using TPN in patients unable to tolerate enteral nutrition.

Previous research has demonstrated that patients at high nutritional risk can benefit from parenteral nutrition, leading to improvements in nutritional status and better weight maintenance (11).

The purpose of this study was to investigate the impact of parenteral nutrition support in HSCT patients at high nutritional risk. This study aimed to provide a detailed analysis of the effects of nutritional support on various clinical outcomes in these patients. By focusing on this specific high-risk population, our study hopes to contribute valuable insights that can help optimize care strategies for individuals undergoing HSCT.

## 2 Methods

### 2.1 Subjects

The Ethics Committee of the Second Hospital of Dalian Medical University approved this retrospective, historically controlled study (approval number: KY2024-170-01). Participants included patients aged 18–68 years who underwent initial HSCT (both autotransplantation and allotransplantation). Of the participants, 80% received allotransplantation and 20% from autotransplantation. The study was conducted at the Department of Hematology in the Second Affiliated Hospital of Dalian Medical University. The inclusion criteria were as follows: (I) patients aged ≥18 years and (II) an NRS-2002 score ≥ 5. The exclusion criteria included (I) complications involving major organ diseases such as cardiac complications (heart failure with a left ventricular ejection fraction (LVEF) ≤ 45%, myocardial infarction, atrial fibrillation or flutter, and sustained ventricular tachycardia), pulmonary complications, hepatic complications [total bilirubin (TB) > 3.49 mg/dL, conjugated bilirubin (CB) > 1.33 mg/dL] (12), or renal complications (without AKI, which is defined as an increased 0.3 mg/dL serum creatinine within 48 h or an increase of 1.5 times baseline within 7 days) (13); (II) ICU requirement (without ICU therapies) (14); and (III) incomplete data records. Based on the NRS-2002 scores, patients were classified into two groups, both with “high nutritional risk” (NRS-2002 scores ≥5) (15).

Patients receiving a mixture of amino acids, fat emulsion, glucose, vitamins, and electrolytes were assigned to the TPN group. Patients who received only glucose injections with electrolytes were assigned to the control group. Glucose and lipids each accounted for 50% of non-protein calories. The TPN was supplemented with electrolytes (including sodium, potassium, calcium, magnesium, and phosphorus), a mixture of hydrosoluble and liposoluble vitamins (including vitamins B, C, A, D, E, K, biotin, and folic acid), and trace elements (including chromium, copper, manganese, selenium, and zinc) (16). Patients in the TPN group received 25–30 kcal·kg^−1^ and 1.0–1.5 g·kg^−1^ of amino acids, whereas patients in the control group received <10 kcal per kilogram of body weight (17).

### 2.2 Source of data

Clinical and TPN-related data were retrieved from electronic medical records and included (I) patient demographics (age, gender, weight, diagnosis, transplant type, hematological data, albumin, and prealbumin), length of hospital stay, and rate of unexpected rehospitalization and (II) TPN prescription details.

### 2.3 Kaplan–Meier curve

Survival analysis was performed using the Kaplan–Meier method, and differences were evaluated with the log-rank test.

### 2.4 Propensity score matching

To minimize selection bias, a 1:1 propensity score matching analysis was performed using the nearest-neighbor method with a caliper of 0.20. The propensity score was calculated based on the following variables: (1) age, (2) sex, (3) NRS-2002 scores, (4) BMI (< 18.5: thin; 18.5 ~ 23.9: normal; 24 ~ 27.9: overweight; ≥28: obesity), (5) diagnosis, (6) transplant type, (7) neutrophil count (NEUT), (8) hemoglobin (HGB), (9) creatinine, (10) glutamic-pyruvic transaminase, (11) glutamic oxaloacetic transaminase, and (12) nutritional risk.

We compared data between the two groups on days 3, 7, 10, and 14 post-transplantation, starting from the day of cell infusion, as well as on the day before discharge, focusing on: (1) effectiveness: weight loss, albumin, and prealbumin; (2) safety: incidence of CRBSI and conjugated bilirubin; and (3) clinical outcomes: length of hospital stay, rate of rehospitalization, hospitalization expenses, and survival rate. The data on body weight were collected every morning before the infusion. A definitive diagnosis of CRBSI was made if the same organism was cultured from at least one percutaneous blood sample and a catheter tip culture or if two blood samples (one from the catheter hub and the other from a peripheral vein) met the criteria for quantitative blood cultures or differential time to positivity (DTP) (18).

### 2.5 Statistical methods

Continuous variables were assessed using the Shapiro–Wilk test and compared using *t*-tests, with results expressed as mean ± standard deviation (SD). Categorical variables were compared using Pearson’s chi-squared test and presented as frequencies. All analyses were performed using SPSS 27.0, with *p* < 0.05 considered statistically significant.

## 3 Results

### 3.1 Patient characteristics

**Figure 1:**
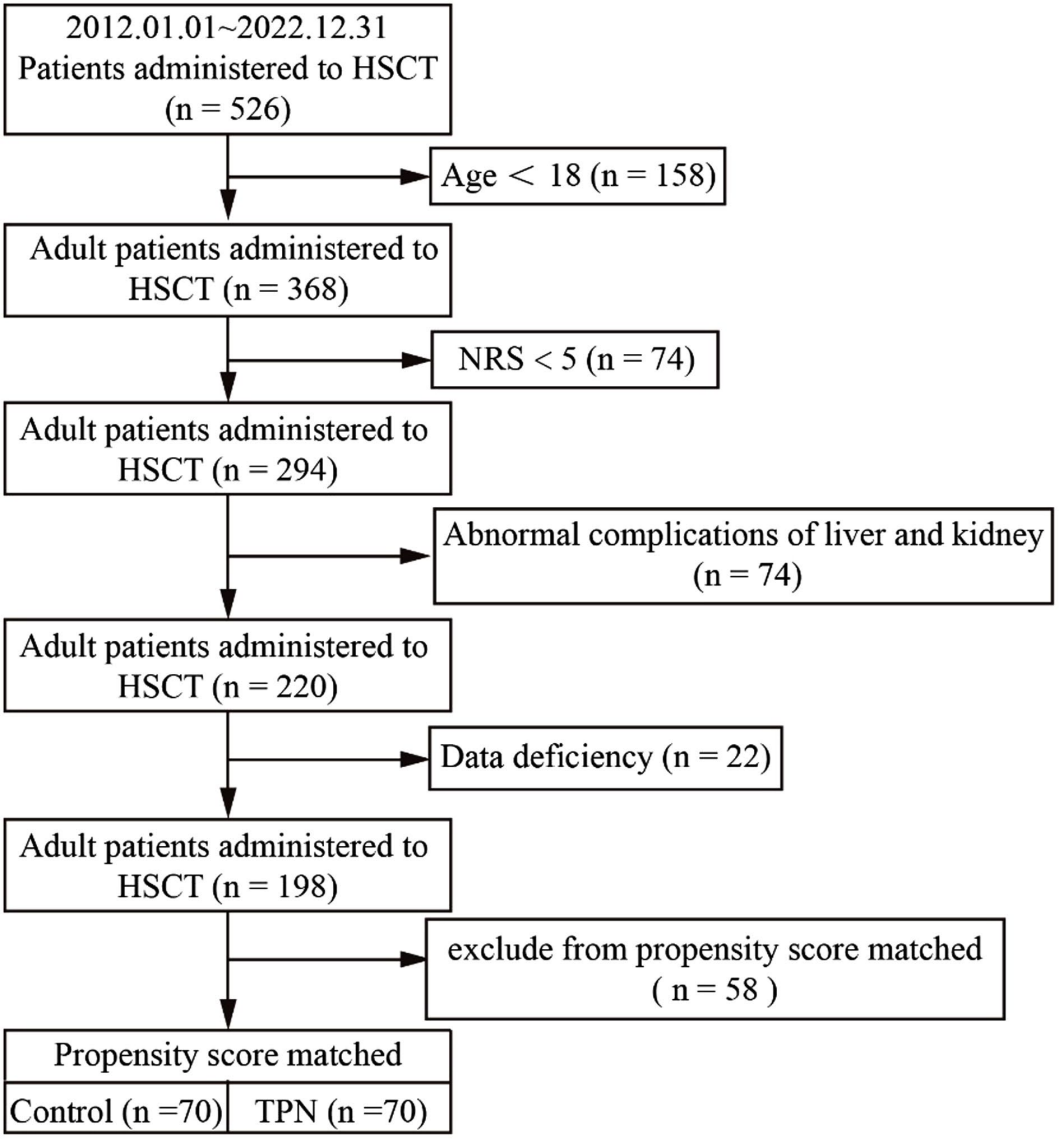
Flowchart of the study population. A total of 526 patients who underwent HSCT between 2012 and 2022 were excluded who were younger than 18 years, with an NRS-2002 score < 5, abnormal liver or kidney function, or missing data, followed by propensity score matching. HSCT, hematopoietic stem cell transplantation; NRS-2002 scores, nutritional risk screening; TPN, total parenteral nutrition.

A total of 526 patients who underwent HSCT between 2012 and 2022 were included in the study. Patients younger than 18 years with an NRS-2002 score < 5, abnormal liver or kidney function, or missing data were excluded from the study, followed by propensity score matching. Figure 1 illustrates the distribution of patients who received or did not receive TPN during the study period.

Before matching, the total bilirubin levels in the TPN group were significantly higher than those in the control group (17.4 mmol/L vs. 12.8 mmol/L; *p* = 0.02). To minimize the effect of baseline nutritional status on outcomes, propensity score matching was conducted, matching 70 patients from the TPN group with 70 patients from the control group. After matching, the total bilirubin levels were similar between the two groups (TPN group: 12.7 mmol/L vs. control group: 14.7 mmol/L; *p* = 0.17), indicating a balanced comparison between the groups (Table 1). Potential factors that could influence TPN outcomes, such as diagnosis, transplant type, and NRS-2002 scores, were also comparable between groups.

**Table 1:** Baseline characteristics of the propensity score-matched patients.

	Control (*n* = 70)	TPN (*n* = 70)	*p*-value
Age (years), (n)	38.26 ± 15.53	38.13 ± 14.19	0.96
Sex (male), (n%)	42	34	0.18
BMI (kg/m^2^)	21.82 ± 3.60	22.10 ± 3.49	0.63
Diagnosis, n (%)			0.65
Aplastic anemia	6 (8.6)	10 (14.3)	
Lymphoma	7 (10.0)	5 (7.1)	
Myeloma	11 (15.7)	11 (15.7)	
Acute lymphoblastic leukemia	13 (18.6)	17 (24.3)	
Acute myeloid leukemia	33 (47.1)	27 (38.6)	
Transplant type, n (%)			0.33
Autotransplantation	15 (21.4)	20 (28.6)	
Allotransplantation	55 (78.6)	50 (71.4)	
NRS-2002 scores, n (%)			0.69
5	17 (24.3)	15 (21.4)	
6	53 (75.7)	55 (78.6)	
NEUT, n (%)			0.65
Normal	59 (84.3)	57 (81.4)	
Abnormal	11 (15.7)	13 (18.6)	
HGB, n (%)			
Normal	63 (90.0)	64 (91.4)	0.77
Abnormal	7 (10.0)	6 (8.6)	
Creatinine, n (%)			0.86
Normal	47 (67.1)	48(68.6)	
Abnormal	23 (32.9)	22(31.4)	
Glutamic-pyruvic transaminase, n (%)			0.32
Normal	70 (100.0)	69 (98.6)	
Abnormal	0 (0.0)	1 (1.4)	
Glutamic oxaloacetic transaminase, n (%)			0.51
Normal	64 (91.4)	66 (94.3)	
Abnormal	6 (8.6)	4 (5.7)	
Total protein, n (%)			0.61
Normal	38 (54.3)	35 (50.0)	
Abnormal	32 (45.7)	35 (50.0)	
Albumin, n (%)			0.59
Normal	48 (68.6)	45 (64.3)	
Abnormal	22 (31.4)	25 (35.7)	
Prealbumin, n (%)			1.0
Normal	70 (100.0)	70 (100.0)	
Abnormal	0 (0.0)	0 (0.0)	
Total bilirubin, n (%)			0.32
Normal	69 (98.6)	70 (100.0)	
Abnormal	1 (1.4)	0 (0.0)	
Conjugated bilirubin			
Normal	37(52.9%)	30(42.9%)	0.61
Abnormal	33(47.1%)	40(57.1%)	

TPN, total parenteral nutrition; BMI, body mass index. Among the measured variables, abnormal HGB < 130 g/L and abnormal neutrophils > 13,000 and < 4,000.

Both groups had similar distributions of diagnosis types: aplastic anemia (8.6% in the control group vs. 14.3% in the TPN group), lymphoma (10.0% vs. 7.1%), and myeloma (15.7% in both groups; *p* = 0.65). The proportion of patients undergoing allotransplantation was also similar between groups (55 patients in the control group vs. 50 in the TPN group), while a smaller number underwent autotransplantation (15 in the control group vs. 20 in the TPN group; *p* = 0.33).

At the time of admission, there were slightly more patients with NRS-2002 scores of 6 or higher in the TPN group compared to the control group; however, this difference was not statistically significant (55 vs. 53; *p* = 0.69).

### 3.2 Outcomes

#### 3.2.1 Effectiveness

**Figure 2:**
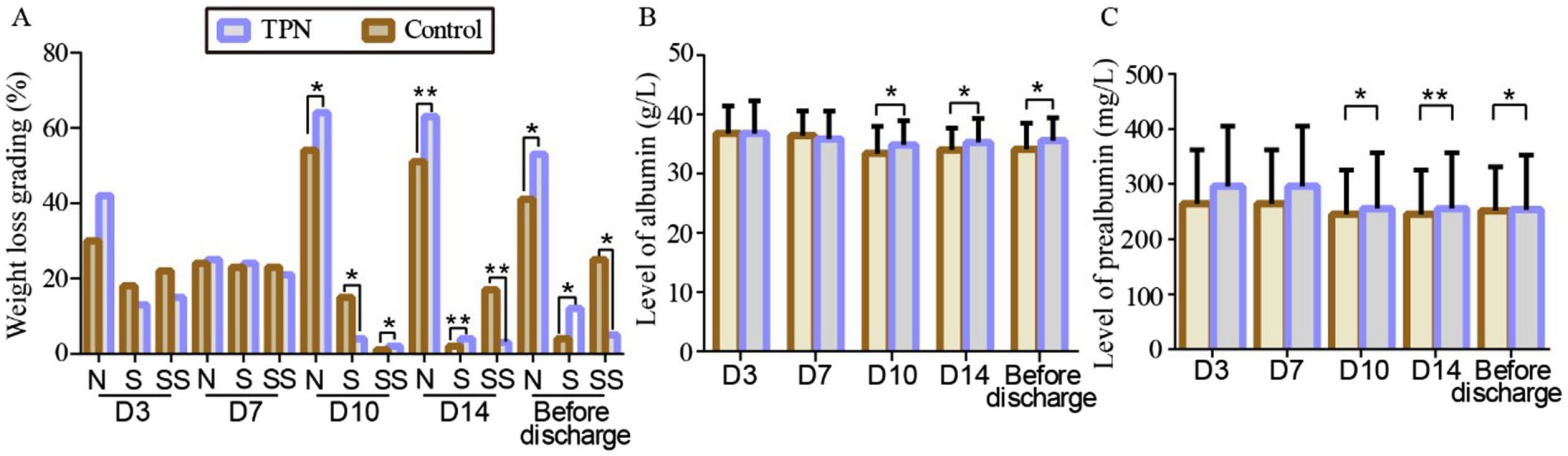
Effect of TPN on the effectiveness of HSCT patients after 3, 7, 10, and 14 days and the day before discharge of transplantation. **(A)** Weight loss grading to N, S, and SS scores of transplant patients. Level of albumin **(B)** and prealbumin **(C)** of transplant patients. N: non-significant weight loss; S: significant weight loss; SS: severe significant weight loss. Compared to the control group, ^**^p < 0.01 or ^*^p < 0.05.

Weight loss was assessed using the Blackburn criteria (19). Clinically severe weight loss in the TPN group (5.7, 5.7, and 17.1%) was significantly less than in the control group (21.4, 24.3, and 35.7%) on day 10, day 14, and the day before discharge, respectively (*p* = 0.024, 0.01, 0.039). The number of patients showing significant weight loss between the non-TPN group (1.4, 2.9, and 5.7%) and the TPN group (2.9, 4.3, and 7.1%) did not differ significantly. The majority of patients experienced non-significant weight loss, with 77.1, 72.9, and 58.6% in the control group and 91.4, 90.0, and 75.7% in the TPN group on day 10, day 14, and the day before discharge, respectively (Figure 2).

TPN’s impact on the nutritional status of transplant patients was also evaluated. On day 10, day 14, and the day before discharge, the TPN group showed a significant increase in albumin and prealbumin levels compared to the control group (*p* < 0.01 or *p* < 0.05) (Figure 2).

#### 3.2.2 Safety

**Figure 3:**
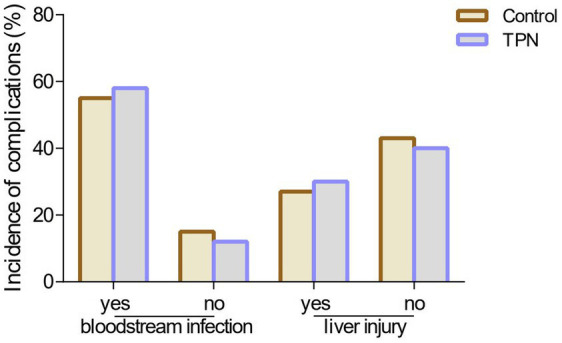
Effect of TPN on the incidence of complications. There were no statistically significant differences in catheter-related bloodstream infection (CRBSI) or conjugated bilirubin levels between the TPN group and the control group.

As presented in Supplementary Tables S5, S6, there were no statistically significant differences in catheter-related bloodstream infection (CRBSI) or conjugated bilirubin levels between the two groups (*p* = 0.52) (Figure 3).

#### 3.2.3 Clinical outcomes

**Figure 4:**
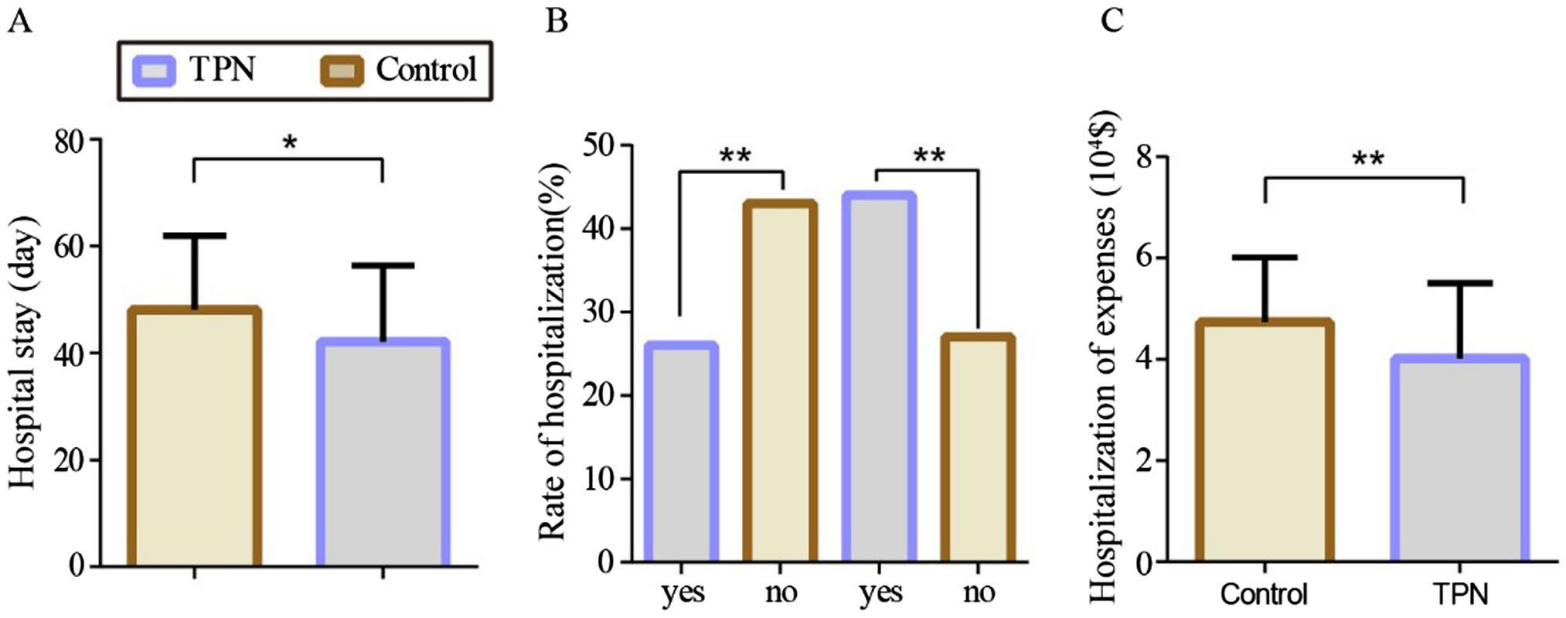
Effect of TPN on the clinical outcome of HSCT patients after 3, 7, 10, 14, and before discharge of transplantation. **(A)** hospital stay, **(B)** rate of hospitalization, and **(C)** hospitalization expenses of HSCT transplant patients. Compared to the control group, ^**^p < 0.01 or ^*^p < 0.05.

The hospital stay for HSCT patients was significantly shorter in the TPN group than in the control group (*p* < 0.05). The rehospitalization rate was also lower in the TPN group than in the control group (*p* < 0.05). Furthermore, the hospitalization expenses in the TPN group were significantly lower than those in the control group (*p* < 0.01) (Figure 4).

**Figure 5:**
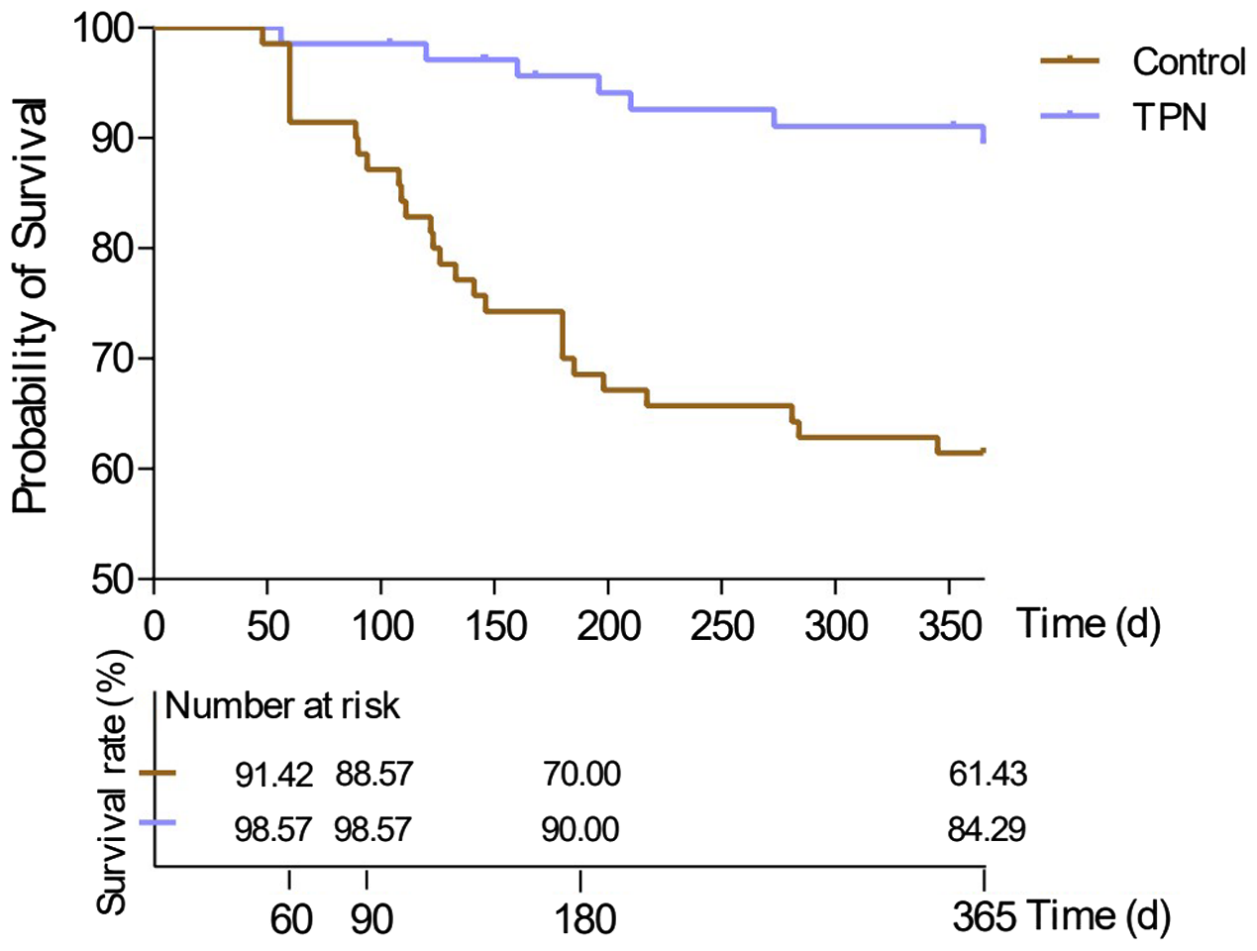
Effect of TPN on the survival of HSCT patients after 2 months, 3 months, 6 months, and 1 year. Patients in the non-TPN group had a shorter overall survival (OS).

In terms of survival rates, the 2-month survival rate was 91% in the non-TPN group and 99% in the TPN group. The 3-month survival rates were 89% vs. 99%, the 6-month survival rates were 70% vs. 90%, and the 1-year survival rates were 61% vs. 84%, respectively. Overall, patients in the non-TPN group had a shorter overall survival (OS) (*p* < 0.01) (Figure 5).

## 4 Discussion

Hematopoietic stem cell transplantation (HSCT) is frequently used to treat hematological malignancies. Patients often receive high-dose chemotherapy or total body irradiation before transplantation, leading to severe side effects such as intense nausea, vomiting, diarrhea, mucositis, and graft-versus-host disease (GVHD), which significantly impair oral intake. In this retrospective study, we evaluated the clinical effects of total parenteral nutrition (TPN) formulations in HSCT patients at high nutritional risk for the first time. Our results suggested that TPN support for these patients improves nutritional status, shortens hospital stays, reduces 1-year post-transplant mortality, and decreases the rate of unexpected rehospitalizations. As TPN support develops, more individualized parenteral nutrition strategies and standardized nutritional care should be implemented.

This study was critical for several reasons. First, previous studies have shown that parenteral nutrition can limit weight loss in HSCT patients (20). We investigated weight loss reduction in patients undergoing HSCT with or without nutritional intervention over a defined period (21). Our findings showed that TPN did not prevent early weight loss after transplantation. In this study, the absence of nutrition support resulted in 21.4 and 24.3% of patients experiencing significant weight loss by day 10 and day 14, respectively, with 35.7% showing considerable weight loss before discharge. TPN intervention reversed these trends, with only 5.7, 5.7, and 17.1% of patients experiencing significant weight loss on day 10, day 14, and the day before discharge, respectively. Weight loss in HSCT patients is caused not only by chemotherapy-induced gastrointestinal toxicity but also by GVHD and the use of immunosuppressive agents (2). Therefore, the primary goal of nutrition support is to prevent malnutrition and deterioration in nutritional status. Our data clearly demonstrated that TPN reduced the proportion of patients with severe weight loss, highlighting the importance of early nutritional intervention for HSCT patients at high nutritional risk.

Another key concern is the risk of catheter-related bloodstream infections (CRBSI) associated with parenteral nutrition (18). Our study found no statistically significant difference in CRBSI rates between the non-TPN and TPN groups. Some studies have suggested that TPN formulations may provide a favorable environment for bacterial growth, increasing the risk of CRBSI (22). In addition, HSCT patients frequently receive parenteral nutrition and blood products through central venous catheters (CVC), which can introduce bacteria or fungi into the bloodstream, leading to CRBSI (23). CRBSI is a serious complication that can prolong hospital stays, increase medical costs, and elevate morbidity and mortality risks (24). While other studies have argued that parenteral nutrition does not increase CRBSI incidence, this may be due to proper CVC care and wound management (25). Our findings emphasize the importance of strict aseptic techniques during TPN preparation, careful catheter care, and minimizing bag changes to mitigate CRBSI risk.

Liver injury is another potential complication in HSCT patients, and parenteral nutrition-associated liver disease (TPNALD) is a common and serious issue (26). TPNALD is usually diagnosed by elevated biochemical markers of liver disease, with conjugated bilirubin levels above 2 mg/dL being a key indicator. Our data showed that TPN did not increase conjugated bilirubin (CB) levels compared to the control group. Although TPN can cause transient increases in aminotransferase concentrations during the first 1–3 weeks, there is no evidence that long-term TPN use leads to TPN-induced liver disease in high-risk patients (27). However, inappropriate ratios in parenteral nutrition formulations can lead to cholestasis or fat accumulation, causing liver damage (TPNALD). Our study underscores the need for regular monitoring of liver function and adjustments to nutritional regimens to prevent liver damage, particularly in HSCT patients. Regular liver function tests and lipid metabolism monitoring are essential when using TPN in this population.

For individuals unable to tolerate enteral nutrition, TPN provides a valuable alternative, ensuring that patients receive adequate calories, amino acids, and essential nutrients. In our study, patients who received TPN before the day of hematopoietic stem cell transfusion, despite being more malnourished, appeared to derive long-term benefits from nutritional support, including shorter hospital stays, reduced 1-year post-transplant mortality, and lower rates of unexpected rehospitalization. Recent research has shown that serum albumin levels are strongly associated with the risk of adverse outcomes (28). Both serum albumin and prealbumin have been used as nutritional markers to assess plasma protein levels, reflecting patients’ overall nutritional status (29, 30). Our results indicated that TPN significantly increased serum albumin and prealbumin levels on day 10, day 14, and the day before discharge. Studies have also demonstrated that low albumin levels, associated with malnutrition, can heighten the risk of adverse outcomes (31). In addition, hypoalbuminemia has been linked to longer mechanical ventilation times, extended hospital stays, and lower survival rates in advanced cancer patients. Prealbumin, with its shorter half-life, can serve as a more sensitive marker for acute nutritional changes (32). At present, a large number of studies have demonstrated the superiority of enteral over parenteral nutrition in bone marrow transplants (33). Conspicuously, this study provides the insight that patients should not be deprived of calories during transplantation since TPN did not increase complication rates. Overall, our TPN intervention improved survival rates and quality of life for patients.

This study is the first to evaluate the clinical outcomes of TPN formulations in HSCT patients at high nutritional risk. TPN intervention significantly mitigated weight loss on day 10, day 14, and the day before discharge, while also boosting key nutritional indicators such as albumin and prealbumin levels. TPN administration also had a favorable impact on CRBSI and conjugated bilirubin levels, reducing rehospitalization rates and hospitalization expenses. Moreover, TPN support improved survival rates at 2 months, 3 months, 6 months, and 1 year after transplantation. Based on these findings, TPN can be safely and effectively used in HSCT patients undergoing induction therapy.

## 5 Limitations

Several limitations should be noted. First, this study was conducted in a single center with a limited number of patients. Second, the retrospective design introduced potential selection biases as we only included data at the time of diagnosis or relapse. In addition, data on dietary intake were limited, and not all patients in the control group received TPN according to strict caloric calculations. Third, a portion of patients in the control group had uncontrolled hyperglycemia and uncontrolled electrolyte abnormalities, and some patients were expected to use parenteral nutrition for no more than 5 days. Therefore, the patients of the control group in our study only received glucose injections with electrolytes.

## 6 Conclusion

Compared to the absence of caloric support, TPN in HSCT patients with high nutritional risk not only improves nutritional status but also significantly shortens hospital stays, reduces 1-year post-transplant mortality, and lowers the rate of unexpected rehospitalizations. However, long-term follow-up is required, and data from large-scale, multi-center studies are needed to validate these findings. Our study offers a novel perspective on enhancing the clinical outcomes of HSCT recipients, particularly those at risk of malnutrition.

## Data Availability

The raw data supporting the conclusions of this article will be made available by the authors, without undue reservation.
