# Supplementary material for: Clinical impacts of total parenteral nutrition in hematopoietic stem cell transplantation patients with high nutritional risk
Source: Front Nutr. 2024 Dec 13;11:1495640. doi: 10.3389/fnut.2024.1495640 (PMC11671266; doi:10.3389/fnut.2024.1495640)
Supplement: Supplementary file 1 [file Data_Sheet_1.docx]

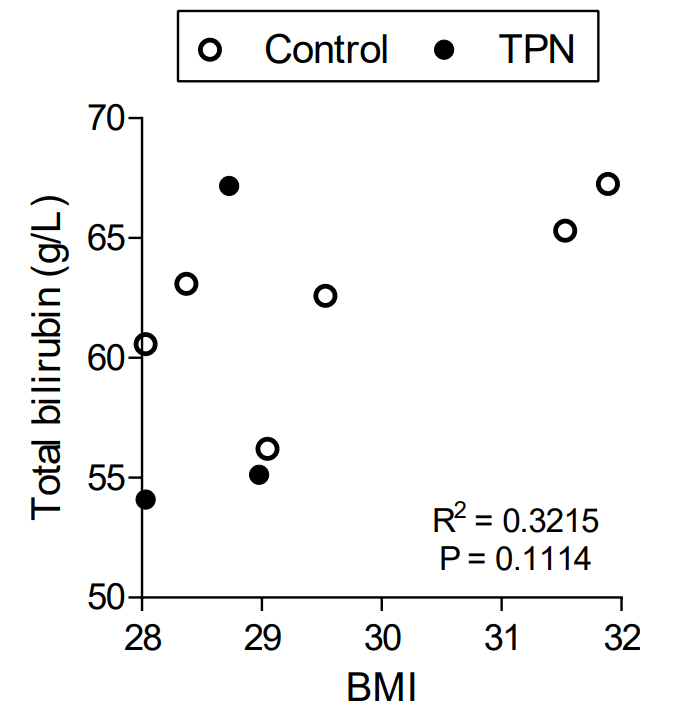


**Supplemental Figure 1** The correlation between the BMI over 28 kg/m^2^ and the total bilirubin of the control and TPN patients after 14 days transfusion.


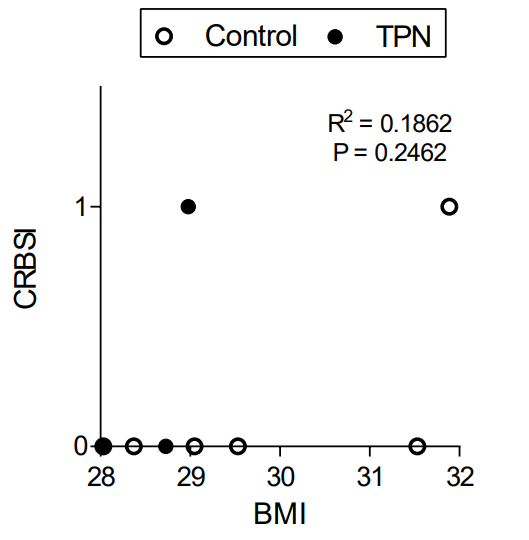


**Supplemental Figure 2** The correlation between the BMI over 28 kg/m^2^ and the CRBSI of the control and TPN patients after 14 days transfusion. "0" indicated no CRBSI, "1" indicated CRBSI.


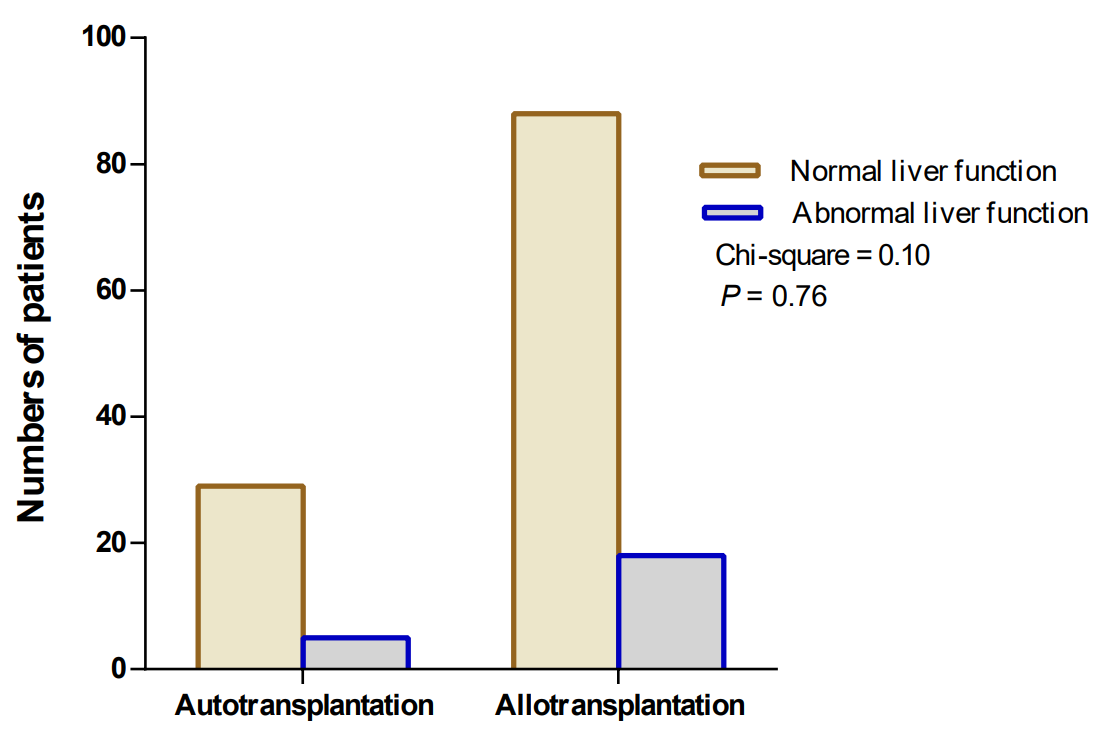


**Supplemental Figure 3** The correlation between the transplantation types and the liver function of the control and TPN patients after 14 days transfusion.


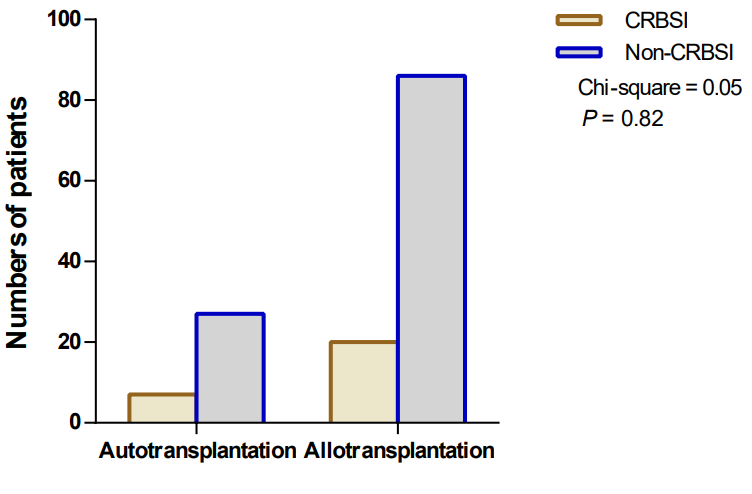


**Supplemental Figure 4** The correlation between the transplantation types and the CRBSI of the control and TPN patients after 14 days transfusion.

**Supplementary Table** **1 Effect of TPN on the weight of transplant patients**

| Time | group | Weight loss (%) | | | sum | χ^2^ | *P* |
| --- | --- | --- | --- | --- | --- | --- | --- |
|  |  | No significance | Significant weight loss | Severe weight loss |  |  |  |
| Day 3 | Control | 30 (42.9%) | 22 (31.4%) | 18 (25.7%) | 70 | 4.131 | 0.127 |
|  | TPN | 42 (60%) | 15 (21.4%) | 13 (18.6%) | 70 |  |  |
| Day 7 | Control | 24 (34.3%) | 23 (32.9%) | 23 (32.9%) | 70 | 0.133 | 0.936 |
|  | TPN | 25 (35.7%) | 21 (30.0%) | 24 (34.3%) | 70 |  |  |
| Day 10 | Control | 54 (77.1%) | 1(1.4%) | 15(21.4%) | 70 | 7.445 | 0.023^*^ |
|  | TPN | 64 (91.4%) | 2(2.9%) | 4 (5.7%) | 70 |  |  |
| Day 14 | Control | 51 (72.9%) | 2(2.9%) | 17(24.3%) | 70 | 9.304 | 0.01^**^ |
|  | TPN | 63 (90.0%) | 3(4.3%) | 4 (5.7%) | 70 |  |  |
| Before discharge | Control | 41 (58.6%) | 4(5.7%) | 25(35.7%) | 70 | 6.473 | 0.045^*^ |
|  | TPN | 53(75.7%) | 5 (7.1%) | 12 (17.1%) | 70 |  |  |

Compared to the control group, ^*^*P* < 0.05

**Supplementary Table 2 Effect of TPN on the albumin of transplant patients**

| Time | N | Control | TPN |
| --- | --- | --- | --- |
| Day 3 | 70 | 36.77±4.64 | 36.78±5.48 |
| Day 7 | 70 | 36.40±4.15 | 35.87±4.66 |
| Day 10 | 70 | 33.41±4.57 | 34.87±4.08^*^ |
| Day 14 | 70 | 33.72±3.52 | 35.27±4.04^*^ |
| Before discharge | 70 | 34.09±4.44 | 35.55±3.87^*^ |

Compared to the control group, ^*^*P* < 0.05

**Supplementary Table 3 Effect of TPN on the prealbumin of transplant patients**

| Time | N | Control | TPN |
| --- | --- | --- | --- |
| Day 3 | 70 | 264.52±97.79 | 296.24±109.12 |
| Day 7 | 70 | 291.28±89.21 | 279.10±102.78 |
| Day 10 | 70 | 245.18±79.94 | 274.26±86.73^*^ |
| Day 14 | 70 | 233.27±79.57 | 279.34±80.20^**^ |
| Before discharge | 70 | 247.24±83.29 | 280.65±100.22^*^ |

Compared to the control group, ^*^*P* < 0.05 or ^**^*P* < 0.01

**Supplementary Table 4 Effect of TPN on the Catheter-related bloodstream infection of transplant patients**

| Time | group | **Catheter-related bloodstream infection** | | | N | | χ^2^ | | *P* | |
| --- | --- | --- | --- | --- | --- | --- | --- | --- | --- | --- |
|  |  | **Yes** | **No** | |  |  |  |  |  |  |
| Before discharge | control | 55 (78.6%) | | 15 (21.4%) | | 70 | | 0.41 | | 0.52 |
|  | TPN | 58 (82.9%) | | 12 (17.1%) | | 70 | |  |  |  |

**Supplementary Table 5 Prevalence of conjugated bilirubin >2 mg/dl in patients under TPN**

| Time | group | **conjugated bilirubin** | | | N | | χ^2^ | | *P* | |
| --- | --- | --- | --- | --- | --- | --- | --- | --- | --- | --- |
|  |  | **Yes** | **No** | |  |  |  |  |  |  |
| Before discharge | control | 27(38.6%) | | 43(61.4%) | | 70 | | 0.27 | | 0.61 |
|  | TPN | 30(42.9%) | | 40(57.1%) | | 70 | |  |  |  |

**Supplementary Table 6 Effect of TPN on the** **hospital stay of transplant patients （‾X±SD）**

| Time | N | Control | TPN |
| --- | --- | --- | --- |
| Before discharge | 70 | 48.06±13.90 | 42.13±14.22^*^ |

**Supplementary Table 7 Effect of TPN on** **the rate for re-hospitalization of HSCT patients**

| Time | group | **rate for**  **re-hospitalization** | | | N | | χ^2^ | | *P* | |
| --- | --- | --- | --- | --- | --- | --- | --- | --- | --- | --- |
|  |  | **Yes** | **No** | |  |  |  |  |  |  |
| Before discharge | control | 26 (37.1%) | | 44(62.9%) | | 70 | | 4.17 | | 0.04^*^ |
|  | TPN | 15(21.4%) | | 55 (78.6%) | | 70 | |  |  |  |

Compared to the control group, ^**^*P* < 0.01

**Supplementary Table 8 Effect of TPN on the** **hospitalization expenses of HSCT patients （‾X±SD）**

| Hospitalization expenses/$ | N | Control | TPN |
| --- | --- | --- | --- |
| Before discharge | 70 | 4.74±1.28 | 4.02±1.49^**^ |

Compared to the control group, ^**^*P* < 0.01
